# Proficiency-based progression training in robot-assisted laparoscopy for endometrial cancer: peri-operative and survival outcomes from an observational cohort study

**DOI:** 10.3389/fmed.2024.1370836

**Published:** 2024-06-05

**Authors:** Ariane Sickinghe, Marielle Nobbenhuis, Ellen Nelissen, Owen Heath, Thomas Ind

**Affiliations:** ^1^Department of Gynecological Oncology, Royal Marsden Hospital, London, United Kingdom; ^2^Faculty of Medicine, University Medical Centre Utrecht, Utrecht University, Utrecht, Netherlands; ^3^Department of Gynecological Oncology, Royal United Hospitals, Bath, United Kingdom

**Keywords:** endometrial cancer, uterus cancer, robot-assisted laparoscopy, minimally-invasive surgery, training, survival, complications

## Abstract

**Introduction:**

Over the last decade there has been a transition from traditional laparoscopy to robotic surgery for the treatment of endometrial cancer. A number of gynecological oncology surgical fellowship programmes have adopted robot-assisted laparoscopy, but the effect of training on complications and survival has not been evaluated. Our aim was to assess the impact of a proficiency-based progression training curriculum in robot-assisted laparoscopy on peri-operative and survival outcomes for endometrial cancer.

**Methods:**

This is an observational cohort study performed in a tertiary referral and subspecialty training center. Women with primary endometrial cancer treated with robot-assisted laparoscopic surgery between 2015 and 2022 were included. Surgery would normally include a hysterectomy and salpingo-oophorectomy with some form of pelvic lymph node dissection (sentinel lymph nodes or lymphadenectomy). Training was provided according to a training curriculum which involves step-wise progression of the trainee based on proficiency to perform a certain surgical technique. Training cases were identified pre-operatively by consultant surgeons based on clinical factors. Case complexity matched the experience of the trainee. Main outcome measures were intra- and post-operative complications, blood transfusions, readmissions < 30 days, return to theater rates and 5-year disease-free and disease-specific survival for training versus non-training cases. Mann–Witney U, Pearson’s chi-squared, multivariable regression, Kaplan-Meier and Cox proportional hazard analyses were performed to assess the effect of proficiency-based progression training on peri-operative and survival outcomes.

**Results:**

Training cases had a lower BMI than non-training cases (30 versus 32 kg/m^2^, *p* = 0.013), but were comparable in age, performance status and comorbidities. Training had no influence on intra- and post-operative complications, blood transfusions, readmissions < 30 days, return to theater rates and median 5-year disease-free and disease-specific survival. Operating time was longer in training cases (161 versus 137 min, *p* = < 0.001). The range of estimated blood loss was smaller in training cases. Conversion rates, critical care unit-admissions and lymphoedema rates were comparable.

**Discussion:**

Proficiency-based progression training can be used safely to teach robot-assisted laparoscopic surgery for women with endometrial cancer. Prospective trails are needed to further investigate the influence of distinct parts of robot-assisted laparoscopic surgery performed by a trainee on endometrial cancer outcomes.

## 1 Introduction

The use of minimally-invasive surgery for endometrial cancer has become widespread since the LACE and GOG LAP2 trials established non-inferiority of laparoscopic versus laparotomic surgery for disease-free and overall survival in endometrial cancer (1–3). Robot-assisted laparoscopic (RAL) surgery was introduced in gynecological surgery in 2005 (4) and provides more precision, better views, reduced patient morbidity and improved surgeon ergonomics compared to conventional laparoscopy enabling the surgeon to perform more complex surgery (5–10). These advantages are especially beneficial in obese patients undergoing open or laparoscopic hysterectomy as they are more prone to post-operative morbidity compared to non-obese patients (11–13). Obesity is the main risk factor for endometrial cancer and since its incidence is rising (14), the preferred approach in minimally-invasive surgery has shifted from straight-stick to robotic (5, 15, 16).

An increasing number of gynecological oncology surgical fellowship programs are embedding RAL surgery. The introduction of a new surgical technique is accompanied with a learning curve, which also has been assessed in conventional laparoscopic and robotic surgery (17–20). This underpins the need for a training curriculum.

Urologists were the first to develop a proficiency-based progression (PBP) training curriculum for robotic surgery (21). The Society of European Robotic Gynecological Surgery and British and Irish Association of Robotic Gynecological Surgeons followed by providing training a curriculum for robot-assisted gynecological surgery (22, 23). Previous studies have evaluated the effect of a single-surgeon learning curve on peri-operative outcomes (11, 13, 14). However, the general effect of PBP training on peri-operative and survival outcomes in endometrial cancer patients undergoing RAL surgery has not been evaluated.

The Royal Marsden Hospital is a tertiary cancer center in the United Kingdom treating high-risk endometrial cancer patients. It was the first center in the United Kingdom to adopt RAL surgery for gynecological cancer in 2007 and have used PBP training in RAL surgery for trainees subspecialising in gynecological oncology since 2015.

The objective of this study was to assess the impact of PBP training on peri-operative and survival outcomes in endometrial cancer patients undergoing RAL surgery.

## 2 Materials and methods

This project received institutional review board approval from the Royal Marsden Committee on Clinical Research on 17-11-2022. Project number SE1234.

### 2.1 Design

An observational cohort study was performed between 2015 and 2022. All patients intended to undergo RAL surgery for endometrial cancer as part of routine care at the Royal Marsden Hospital were included. This included a small group of patients who were operated in any other hospital of the Southwest Thames Gynecological Cancer Centre our surgical team operated in due to capacity constraints. The Southwest Thames Gynecological Cancer Centre is a consortium of hospitals that closely work together and share facilities. It includes the Royal Marsden Hospital, St George’s Hospital, The London Clinic, Lister Hospital and the Princess Grace Hospital. All surgeries were performed by three robot-trained gynecological oncology surgeons on three generations of Da Vinci robots (S, Si, Xi).

The inclusion criteria consisted of women diagnosed with primary endometrial cancer and the intention of undergoing RAL hysterectomy, bilateral salpingo-oophorectomy and/or any lymph node dissection. All subtypes of endometrial cancer were included. Patients with any additional cancer, e.g., simultaneous ovarian, sigmoid or other type of cancer, were excluded from analysis. Patients who underwent RAL hysterectomy, bilateral salpingo-oophorectomy and/or any lymph node dissection for a non-endometrial type of cancer, e.g., cervical cancer, were excluded. Also, conforming to European Society of Gynecological Oncology (ESGO) guidelines (24) patients with advanced disease where cytoreduction was considered infeasible as judged by a multi-disciplinary team were excluded.

### 2.2 Data collection

Data was collected prospectively by two surgeons (TI and MN) from 2015 to 2022 and was stored in an encrypted and secure database. Missing data was completed retrospectively by independent researcher (AS) in 2022 using information on the hospital’s electronic patient record.

### 2.3 Identification of training cases

Trainees subspecialising in gynecological oncology were consultant surgeons subspecialising in gynecological oncologic surgery or gynecological registrars following the training program at the end of general gynecology training. Trainees followed a PBP training curriculum during 1–2 years provided by The Society of European Robotic Gynecological Surgery and British and Irish Association of Robotic Gynecological Surgeons (22, 23). Recommendations for this training curriculum were formulated by experienced gynecological robotic surgeons who performed The Delphi process (25, 26).

In PBP training, trainees follow a structured and standardized training with pre-set learning goals. Modules of training lead from e-learning, to virtual training, to model training, to procedural training. All clinical procedures are performed under the guidance of expert tutors and trainees can only progress to the next step of training if they are proficient in the previous steps as judged by supervising consultant surgeons in concordance with the SERGS training curriculum (22). Trainees start procedural training with vault suturing and end with performing a hysterectomy and lymph node dissection independently.

A case was marked as a “training case” if the trainee performed a part of the surgery on the console. A case was not marked as a “training case” if the trainee only performed first or second assistant tasks like robot docking, skin suturing or bringing in the uterus manipulator. PBP training cases were identified pre-operatively by consultant surgeons based on clinical factors, such as BMI and comorbidities. Patients with a BMI > 50 or many comorbidities were not selected as training cases. Training case complexity was subjectively matched by the supervising surgeon to the trainee’s proficiency and progression through the training program in concordance with the SERGS training curriculum (22).

All operations were performed at the Southwest Thames Gynecological Cancer Centre under direct supervision of one of two consultant surgeons. Consultant surgeons had extensive experience in robotic surgery (over 300 robotic cases performed per surgeon) and were trained by Lapco to provide training in minimally invasive surgery in a similar and certified manner (27).

### 2.4 Outcomes

Primary outcomes included intra- and post-operative complications before and after 30 days, blood transfusions, readmissions < 30 days, return to theater, and 5-year disease-free and disease-specific survival. Intra-operative complications were defined as any type of surgical complication occurring during the operation. Post-operative complications within 30 days were graded according to the Clavien-Dindo classification (28).

Secondary outcomes included estimated blood loss, operating duration, rate of conversions, critical care unit (CCU)-admissions, length of stay (LOS) longer than one day and lymphoedema. A conversion was defined as the need to convert to laparotomy after docking of the robot due to an intra-operative complication or impossibility to complete robotically.

Prognostic risk groups according to ESGO/The European Society for Radiotherapy and Oncology (ESTRO)/The European Society of Pathology (ESP) were assessed (“ESGO risk groups” in short) (24). These recently developed guidelines for risk group determination incorporate clinicopathologic and molecular parameters and effectively predict survival in endometrial cancer (29).

### 2.5 Statistical analysis

Analyses were performed with the Statistical Package for the Social Sciences (SPSS) 28.01.1. Missing data analysis revealed missing data > 5% for American Society of Anesthesiologists physical (ASA) status classification and World Health Organization (WHO) performance status. Imputation of missing data was done in SPSS using the median of nearby points for the variables ASA status classification and WHO performance status (30).

Mann–Witney U testing was used to assess differences in median values. Pearson’s chi-squared testing was performed to assess the correlation between categorical dependent variables and the independent variable (training case yes/no). Multivariable logistic regression analysis was performed to assess the correlation between continuous clinical variables and the independent variable. Multivariable logistic regression analysis was performed to assess the correlation between training and peri-operative outcomes. Cox-regression analysis was performed for 5-year disease-free and disease-specific survival.

The effect of PBP training on post-operative and survival outcomes are expressed as odds and hazard ratios. Effect sizes were corrected for age, stage (< 2/≥ 2) and grade (low/high) analysis because these variables render clinical relevance for disease-free and disease-specific survival.

Statistical tests were two-sided with significance set at *p* < 0.05, with confidence intervals (CI) at the 95% level. Post-hoc testing according to Bonferroni was performed if Pearson’s chi-squared testing rendered group differences (31). Bonferroni-corrected *p*-values are marked with an “*”.

## 3 Results

### 3.1 Patient characteristics

In total 594 endometrial cancer cases were analyzed: 294 (49.4%) training cases and 300 (50.6%) non-training cases. Thirteen cases (1.9%) were excluded due to non-endometrial primary histology or any additional cancer. Eighteen gynecological oncology trainees were trained in a PBP manner with a mean number of 16 cases performed per trainee (range: 4–58).

Table 1 shows the baseline characteristics for training and non-training cases. Groups were similar in age (66 versus 67 years, *p* = 0.095), median ASA physical status score (2 versus 2, *p* = 0.655), median WHO performance status (1 versus 1, *p* = 0.589) and Carlson Comorbidity Index 10-year median survival estimates (21.4 versus 21.4%, *p* = 0.259). Training cases had a lower BMI than non-training cases (30 versus 32 kg/m^2^, *p* = 0.013).

**TABLE 1:** Patient characteristics.

	Training case (*n* = 294)	Non-training case (*n* = 300)	*p*-value
Age (years), median (range)	66 (31–91)	67 (34–93)	0.980
BMI (kg/m2), median (range)	30 (16–57)	32 (17–69)	0.013
ASA score, median (range)	2 (0–3)	2 (1–3)	0.655
**ASA score, *n* (%)**			
0	1 (0.4)	0	
1	29 (10.5)	28 (9.8)	
2	152 (54.9)	156 (54.5)	
3	95 (34.3)	102 (35.7)	
WHO performance status, median (range)	1 (0–3)	1 (0–3)	0.589
**WHO performance status, *n* (%)**			
0	77 (28.4)	100 (35.2)	
1	154 (56.8)	128 (45.4)	
2	30 (11.1)	32 (11.3)	
3	10 (3.7)	22 (7.7)	
Charlson Comorbidity Index 10-year survival estimate, median (range)	21.4% (0–90.1%)	21.4% (0–98.3%)	0.259
FIGO stage, median (range)	1 (1–4)	1 (1–4)	0.224
**FIGO stage, *n* (%)**			
1	190 (64.6)	219 (73.0)	
2	25 (8.5)	20 (6.7)	
3	68 (23.1)	52 (17.3)	
4	11 (3.7)	9 (3.0)	
**Histology, *n* (%)**			
Endometrioid	167 (56.8)	208 (69.3)	0.002*
Serous	69 (23.5)	54 (18.0)	0.101*
Clear cell carcinoma	14 (4.8)	9 (3.0)	0.267*
Carcinosarcoma	26 (8.8)	19 (6.3)	0.246*
Other	18 (6.1)	10 (3.3)	0.110*
Grade, median (range)	3 (1–3)	2 (1–3)	0.004
**Grade, *n* (%)**			
1	100 (34.0)	136 (45.3)	0.004*
2	46 (15.6)	45 (15.0)	0.826*
3	148 (50.3)	119 (39.7)	0.009*
**ESGO Risk, *n* (%)**			0.069
Low	70 (24.3)	95 (31.9)	
Intermediate	51 (17.7)	60 (20.1)	
High-Intermediate	29 (10.1)	34 (11.4)	
High	131 (45.5)	101 (33.9)	
Advanced	7 (2.4)	8 (2.7)	
**Type of LN dissection, *n* (%)**			
Sentinel LN dissection	227 (77.2)	203 (67.7)	0.009
Pelvic lymphadenectomy	74 (25.2)	88 (29.3)	0.255
Para-aortic lymphadenectomy	1 (0.3)	4 (1.3)	0.185
LN harvested, median (range)	3 (0–37)	3 (0–39)	0.337
**(Neo-)adjuvant treatment, *n* (%)**			
Neo-adjuvant treatment	15 (5.1)	11 (3.7)	0.253
Adjuvant treatment	197 (69.9)	170 (59.4)	0.031
Follow-up duration (months), median (range)	25 (0–60)	28 (0–60)	0.148

ASA, American Society of Anesthesiologists; BMI, body mass index; ESGO, the European Society of Gynecological Oncology; FIGO, International Federation of Gynecology and Obstetrics; LN, lymph node; WHO, World Health Organization.

*Post-hoc analyses were performed for WHO performance status, histology and grade and *p*-values were adjusted following the Bonferroni method (25).

No differences were found in median Fédération Internationale de Gynécologie et d’Obstétrique (FIGO) stage between groups (1 versus 1, *p* = 0.224), but training cases had patients with a higher median histopathological grade (3 versus 2, *p* = 0.004). Post-hoc testing showed a lower rate of grade 1 (34.0 versus 45.3%, *p* = 0.004*) and a higher rate of grade 3 tumors (50.3 versus 39.7%, *p* = 0.009*) in training cases. Training cases had a lower rate of endometrioid tumors (56.8 versus 69.3%, *p* = 0.002*) and a higher percentage of adjuvant treatment in training cases (69.9 versus 59.4%, *p* = 0.031). The distribution of European Society of Gynecological Oncology (ESGO) risk scores (29) did not differ between groups (*p* = 0.069). More sentinel lymph node dissections (77.2 versus 67.7%, *p* = 0.009) were performed in training cases. The median number of harvested lymph nodes (3 versus 3, *p* = 0.337), rates of pelvic (25.2 versus 29.3%, *p* = 0.255) and para-aortic lymphadenectomies (0.3 versus 1.3%, *p* = 0.185) were comparable. Median follow-up was comparable between groups (25 versus 28 months, *p* = 0.148).

### 3.2 Primary outcomes

**FIGURE 1:**
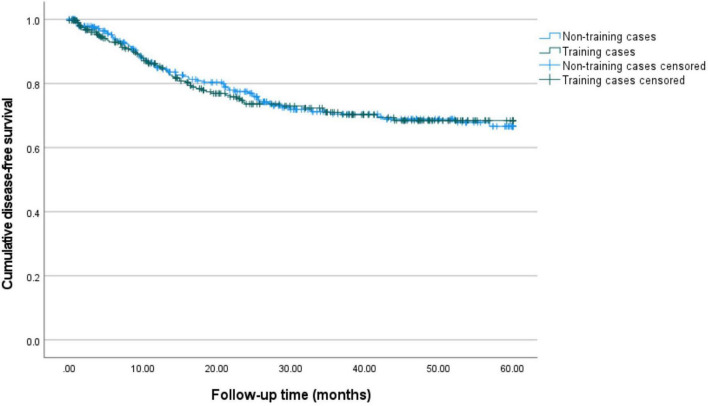
5-year disease-free survival for training and non-training cases. Kaplan–Meier curves for 60 months of follow-up are presented. Training cases are depicted in green and non-training cases are depicted in blue. The estimated 5-year disease-free survival is 66.6% (95%-CI: 59.1–73.0%) for non-training cases and 68.5% (95%-CI: 61.3–74.5%) for training cases.

**FIGURE 2:**
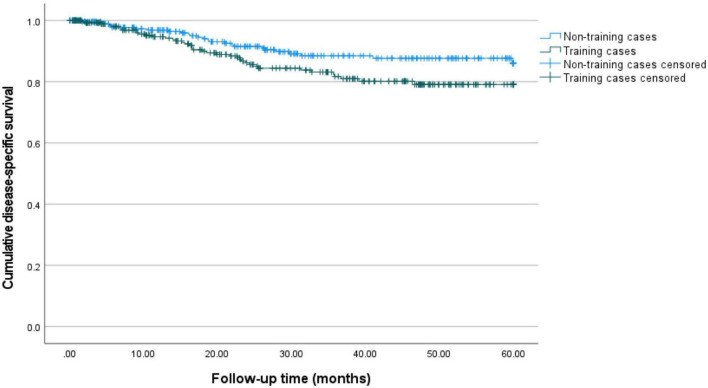
5-year disease-specific survival for training and non-training cases. Kaplan–Meier curves for 60 months of follow-up are presented. Training cases are depicted in green and non-training cases are depicted in blue. The estimated 5-year disease-specific survival is 86.1% (95%-CI: 80.0–90.6%) for non-training and 79.1% (95%-CI: 72.6–84.2%) for training cases.

Primary outcomes are displayed in Tables 2, 3 and Figures 1, 2.

**TABLE 2:** Peri-operative and survival outcomes in training and non-training cases.

	Training case (*n* = 294)	Non-training case (*n* = 300)	*p*-value
EBL (ml), median (range)	100 (0–1,200)	100 (0–2,700)	0.005
Conversion, *n* (%)	7 (2.4)	6 (2.0)	0.749
Blood transfusion, *n* (%)	6 (2.0)	14 (4.7)	0.076
Operating time (min), mean (range)	160 (25–308)	137 (20–385)	< 0.001
CCU-admission, *n* (%)	7 (2.5)	10 (3.6)	0.991
Length of stay (days), median (range)	1 (0–77)	2 (1–30)	0.007
Intra-operative complication, *n* (%)	14 (4.8)	23 (7.7)	0.143
Post-operative complication, *n* (%)	84 (30.4)	86 (30.4)	0.991
**Clavien-Dindo grade, *n* (%)**			0.665
1	30 (10.9)	21 (7.4)	
2	48 (17.4)	59 (20.8)	
3a	2 (0.7)	1 (0.4)	
3b	2 (0.7)	3 (1.1)	
4a	2 (0.7)	2 (0.7)	
Readmission < 30 days, *n* (%)	11 (3.8)	18 (6.0)	0.205
Return to theater, *n* (%)	3 (1.0)	5 (1.7)	0.492
Lymphoedema, *n* (%)	25 (8.4)	43 (11.3)	0.192
Disease-specific survival (months), median (range)	21 (0–60)	26 (0–60)	0.004
Disease-free survival (months), median (range)	25 (0–60)	28 (0–60)	0.182
Disease-specific death, *n* (%)	38 (12.8)	36 (9.1)	0.178
Recurrence, *n* (%)	66 (24.2)	62 (25.1)	0.825

CCU, critical care unit; EBL, estimated blood loss. Data are presented as *n* (% of training cases/non-training cases, rounded to one decimal).

**TABLE 3:** Multivariable logistic regression on the effect of training on post-operative outcomes and cox proportional hazard ratios for disease-free and disease-specific survival.

	Odds ratio (95%-CI)	*p*-value
Intra-operative complication	0.7 (0.4–1.4)	0.364
Clavien-Dindo complication	0.9 (0.6–1.2)	0.436
Any post-operative complication	0.9 (0.6–1.2)	0.385
Readmission < 30 days	0.7 (0.3–1.5)	0.332
Return to theater	0.7 (0.2–2.8)	0.579
LOS > 1 day	0.6 (0.5–0.9)	0.003
Lymphoedema	0.6 (0.4–1.1)	0.101
	**Hazard ratio (95%-CI)**	***p*-value**
Disease-free survival	0.9 (0.6–1.4)	0.892
Disease-specific survival	1.5 (0.8–2.6)	0.172

CI, confidence interval; LOS, length of stay. Odds and hazard ratios are corrected for stage, grade and age. Stage was divided in stage 1 or ≥ 2. Grade was divided in low (grade 1 and 2) and high grade (grade 3). Age was categorized per five years.

#### 3.2.1 Peri-operative outcomes

Intra- and post-operative complications were comparable across groups (4.8 versus 7.7%, *p* = 0.146; 30.4 versus 30.4%, *p* = 0.991, respectively). There was no difference in the distribution of Clavien-Dindo complication grades between groups (*p* = 0.665). Readmissions < 30 days (3.8 versus 6.0%, *p* = 0.205), return to theater (1.0 versus 1.7%, *p* = 0.492) and blood transfusions (2.0 versus 4.7%, *p* = 0.076) did not differ (see Table 2).

The effect of PBP training on intra- and post-operative outcomes are expressed as odds and hazard ratios and shown in Table 3. Training did not increase odds ratios for intra-operative complications (0.6, *p* = 0.154), post-operative complications (1, *p* = 0.994), Clavien-Dindo complications, (0.9, *p* = 0.583), readmissions < 30 days (0.6, *p* = 0.202) and return to theater (0.6, *p* = 0.553).

#### 3.2.2 Survival outcomes

Table 2 displays the rates of recurrences (24.2 versus 25.1%, *p* = 0.825) and deaths of disease (12.8 versus 9.1%, *p* = 0.178), which did not differ between groups. Median disease-free survival differed significantly between training and non-training cases (21 versus 26 months, *p* = 0.004). Median disease-specific survival (25 versus 28 months, *p* = 0.182) was comparable between groups. After correction for the confounders age, stage and grade (see methods) the hazard ratio for disease-free survival for training cases compared to non-training cases is 0.9 (95%-CI: 0.6–1.4, *p* = 0.892). For disease-specific survival the hazard ratio is 1.5 (95%-CI: 0.8–2.6, *p* = 0.172) after correction (see Table 3).

Figures 1, 2 show the Kaplan-Meier curves for 5-year disease-free and disease-specific survival. The estimated 5-year disease-free survival is 66.6% (95%-CI: 59.1–73.0%) for non-training cases and 68.5% (95%-CI: 61.3–74.5%) for training cases. The estimated 5-year disease-specific survival is 86.1% (95%-CI: 80.0–90.6%) for non-training and 79.1% (95%-CI: 72.6–84.2%) for training cases.

### 3.3 Secondary outcomes

Secondary outcomes are displayed in Tables 2, 3. A difference was found in the range of estimated blood loss (0–2,700 ml versus 0–1,200 ml, *p* = 0.005) favoring training cases. This did not result in a difference in blood transfusions (as stated above). The rates of conversions (2.4 versus 2.0%, *p* = 0.749) and CCU-admissions (2.5 versus 3.9%, *p* = 0.991) were comparable. Mean operating time was found to be longer in training cases (160 min versus 137 min, *p* ≤ 0.001). Lymphoedema rates did not differ between groups (8.4 versus 11.3%, *p* = 0.192). LOS was shorter in training cases (1 day versus 2 days, *p* = 0.007).

Odds and hazard ratios for secondary outcomes are shown in Table 3. Training did not increase the odds ratio for lymphoedema (0.6, *p* = 0.110). Training cases had a lower odds for LOS > 1 day (0.6, *p* = 0.004).

## 4 Discussion

### 4.1 Summary of main findings

PBP training had no impact on intra- and post-operative complications, blood transfusions, readmissions < 30 days, return to theater rates and 5-year disease-free and disease-specific survival in RAL surgery for endometrial cancer. Therefore, it can be safely used as a training method for robotic surgery. As expected, operating time was longer in training cases but this did not have a detrimental effect on patient outcome.

### 4.2 Interpretation of results

We found a significantly shorter median LOS and a lower odds for LOS > 1 day in training cases compared to non-training cases. This might be associated with a gradual increase in the amount of training cases over time (37.2% in 2015 versus 55.3% in 2022) and a simultaneous slight decrease in LOS over time (2 days in 2015 versus 1 day in 2022) due to changed surgical protocols. We found a similar trend in sentinel lymph node procedures. More sentinel lymph node dissections were performed in training cases, which is possibly associated with the gradual increase in training cases over time (37.2% in 2015 versus 55.3% in 2022) accompanied with the simultaneous increase in sentinel lymph node procedures (39.5% in 2015 versus 72.4% in 2022).

In our data median disease-free survival differed between training and non-training cases. However, we also found a difference in tumor grades between training and non-training cases with a lower rate of grade 1 and higher rate of grade 3 tumors in training cases. Grade, stage and age are known predictors for endometrial cancer survival (14). Hence, we corrected for these confounders using multivariate regression analysis. After correction no influence of PBP training on disease-free and disease-specific survival was found.

### 4.3 Results in the context of published literature

To date no other studies have evaluated the general effect of PBP training in RAL surgery for endometrial cancer on peri-operative and survival outcomes. However, the effect of a learning curve for RAL surgery in endometrial cancer on peri-operative outcomes has been identified by two single-surgeon studies (32, 33). By comparing peri-operative outcomes between cases performed in the early stages of the learning curve and cases performed in later stages of the learning curve, we can roughly compare these results with our training and non-training cases. However, it must be noted that these studies were performed by single surgeons and only assessed a limited number of peri-operative outcomes.

One study (32) observed less estimated blood loss in cases performed early in the learning curve compared to later cases, which was also observed in our cohort. BMI is a possible confounder of EBL, with more blood loss and more blood transfusions in higher BMI groups (34). Since BMI was significantly higher in non-training cases, this is another possible explanation for the significant difference in EBL although no difference in blood transfusion rates was found. Our results on operating time are in line with two other studies (32, 33), that found significant improvements in operating time between cases performed in early stages of the learning curve and later cases.

Obese patients undergoing laparoscopic surgery are more prone to surgical and post-operative complications compared to non-obese patients (11–13, 35). Therefore, previous studies on surgical outcomes in endometrial cancer have performed case-matching based on BMI (36). On the other hand, a recent study by Uwins et al. (34) on surgical outcomes of robotic surgery for endometrial cancer did not perform matching on BMI and found no negative influence of BMI on hospital stay and conversion rate. In our study BMI differed significantly between training and non-training cases and this might have been a confounding factor for intra- and post-operative outcomes. However, additional univariate analysis showed no influence of BMI on intra- and post-operative outcomes (data not shown).

No studies assessing the learning curve of robot-assisted laparoscopic surgery for endometrial cancer evaluated survival outcomes. However, Baeten et al. (17) assessed 5-year disease-free and disease-specific survival for cervical cancer patients undergoing RAL surgery and found worse outcomes for cases in early stages of the learning curve compared to cases in later stages. Comparable results were found by two more studies (19, 20). We did not find such a trend in our cohort, which might be due to several differences with our study. First, whereas the previously mentioned studies (17, 19, 20) analyzed cases between 2007 and 2018 when there was no set training curriculum, we analyzed cases between 2015 and 2022 in which timeframe PBP training was implemented. Secondly, we did not look into individual learning curves as Baeten et al. (17) did but investigated the overall effect of PBP training on survival outcomes, which renders the possibility of underestimation of our survival outcomes (see limitations). Lastly, the effects of training in RAL surgery might differ between cervical and endometrial cancer.

In 2020 a new guideline for the definition of prognostic risk groups in endometrial cancer was formulated by ESGO/ESTRO/ESP (24). These guidelines incorporate clinicopathological with molecular variables, e.g., p53 and POLE mutation status, and effectively predict survival in endometrial cancer patients (29). Since then local protocols have been updated, but regional disparities in adherence to the guidelines still exist. This needs to be overcome to decrease the use of adjuvant therapies to spare morbidity (37, 38). Radiomics, the field in which a large number of quantitative features from radiological images are analyzed using data-characterization algorithms, is another field that potentially has an added value for the prediction of prognosis for endometrial cancer patients (39).

During our study the ESGO/ESTRO/ESP guidelines were published and our protocols were updated and implemented. However, as this implementation took its time we did not perform molecular analysis for all cases and treatment protocols were being adjusted during our study. Therefore, we chose to assess the risk groups according to the new guidelines to increase comparability with similar cohorts, but not correct for them in our main analysis. Additional analysis showed no impact of training on disease-free and disease-specific survival after correction for ESGO risk groups (data not shown).

Due to a limited number of studies in RAL surgery in gynecological oncology, we looked at other fields of robotic surgery to compare our results. A PBP training curriculum for robotic-assisted radical cystectomy by the European Association of Urologists Robotic Urology Section was recently evaluated (40). As in our cohort, operating time was significantly longer in training cases, but otherwise the trainee showed non-inferiority compared to the experienced surgeon in terms of estimated blood loss, positive soft tissue margins, number of resected lymph nodes, overall and high-grade complications, and 90-day readmissions.

Lastly, our results are in line with a meta-analysis including 19 randomized controlled trials comparing peri-operative and survival outcomes between trainees and experts in laparotomic and laparoscopic colorectal surgery. They observed a longer operating time in training cases and found no difference in survival outcomes for oncological surgery between trainees and experts (41).

So, considering all literature described above the results of our study are within expectations.

### 4.4 Strengths and weaknesses

Our study has several strengths. First, all procedures were performed by one surgical team in a high-volume tertiary cancer center service resulting in a large cohort with highly comparable surgical circumstances. Moreover, all consultant surgeons had extensive experience in robotic surgery (over 5 years) before subspecialty training was provided and consultant surgeons were trained to provide training in a certified manner (27). Secondly, whereas previous studies have evaluated the performance of only one or two trainees, our study includes a cohort of 18 trainees (32, 33, 40). This makes our results robust and generalizable. Moreover, our results reflect a real-world training setting in an experienced training center. This makes our results likely to be applicable to other training centers. Thirdly, our data was collected prospectively which reduces the chance of information bias and results in a limited amount of missing data. One independent researcher completed the database retrospectively thereby further reducing the likelihood of information bias.

The main limitation of our study is that we did not record which part of the surgery was performed by the trainee. Thereby we were unable to define the effect of performance of specific parts of the surgery by a trainee on peri-operative and survival outcomes possibly underestimating the effect of training in individual steps of RAL surgery on our outcomes. On the other hand, our results highlight a real-world training environment and show some expected differences between training and non-training cases (lower BMI and longer operating time) suggesting that our study has the distinguishing capacities needed to pick up major differences between training and non-training cases.

Compared to other robotic cohorts (34, 36, 42) we have a high grade/high stage cohort which is related to the tertiary referral status of our department. This might limit the generalizability of our results. Direct comparison with other robotic cohorts is needed to further evaluate the effect of PBP training on peri-operative and survival outcomes for RAL surgery in all stages of endometrial cancer.

### 4.5 Implications for practice and future research

Our results show that PBP training can be used safely to teach RAL surgery for endometrial cancer in a high-volume tertiary cancer service with no difference in peri-operative and survival outcomes. We suggest that a PBP training curriculum for RAL surgery should be implemented in gynecological oncology fellowships. We aim to design prospective trials to further investigate the influence of distinct parts of RAL surgery performed by a trainee on peri-operative and survival outcomes.

## Data availability statement

The raw data supporting the conclusions of this article will be made available by the authors, without undue reservation.

## Ethics statement

The studies involving humans were approved by the Clinical Research Committee of the Royal Marsden Hospital. The studies were conducted in accordance with the local legislation and institutional requirements. The ethics committee/institutional review board waived the requirement of written informed consent for participation from the participants or the participants’ legal guardians/next of kin because of the use of patient data in a research setting.

## Author contributions

AS: Writing – original draft, Writing – review & editing. MN: Writing – original draft, Writing – review & editing. EN: Writing – original draft, Writing – review & editing. OH: Writing – original draft, Writing – review & editing. TI: Writing – original draft, Writing – review & editing.
